# What people say # what people do

**DOI:** 10.1007/s40037-015-0163-2

**Published:** 2015-01-17

**Authors:** Jeroen J. G. van Merrienboer

**Affiliations:** Maastricht University, School of Health Professions Education, PO Box 616, 6200 MD Maastricht, The Netherlands

I am writing this letter because I am worried about the increasing use of subjective data in the field of Health Professions Education, including publications in *Perspectives on Medical Education*. Let me first state that there is nothing wrong with the use of subjective data as such. It is clearly the best way to go for answering research questions dealing with opinions, perceptions and feelings. Asking people ‘what they think’, ‘how they see things’ and ‘how they feel’ then yields valuable data. But it is wrong to think that all research questions can be reliably answered on the basis of such subjective data. When we are interested in behaviour and cognitive processes, subjective data are often not reliable and may even be misleading. Let me give three reasons for this.

First, participants are often inclined to ‘please the experimenter’. As soon as an experimenter sits in a room with his or her participants, gives them a task and watches them intently, they will start saying what they think the experimenter expects from them. The characteristics of the experimenter may also influence how participants will act during the experiment, a phenomenon known as ‘experimenter bias.’ This bias might even have a biological basis. For example, in one study participants were asked to rate their pain tolerance and pain unpleasantness [1]. When the experimenter was a high-status university professor they indicated higher pain tolerance and lower pain unpleasantness than when the experimenter was a low-status research assistant. Interestingly, blood pressure reactivity mediated the relation between experimenter status and pain tolerance, suggesting that the higher status of the experimenter elicits physiological adaptations that reduce sensitivity to pain.

Second, researchers may easily forget that the relationship between attitudes and behaviour is weak [2]. When we ask people what they think about separating waste (separate disposal of paper, glass, plastic etc.), many of them will say they are in favour of it. And when we ask people what they think of wind energy, again many of them will say they are in favour of it. This is really their attitude, meaning that they are not ‘lying’ to the experimenter but giving their trustworthy opinion. Yet, we would be making a big mistake if we concluded that these people will indeed separate their waste or accept a wind turbine in their neighbourhood. Many of the people who are in favour of separating waste will not do so, simply because it is not part of their daily behavioural routines. And many of the people who are in favour of wind energy may protest when a wind turbine is planned in their own neighbourhood, something called the NIMBY syndrome (Not In My Back Yard).

Third, many cognitive processes are largely unconscious or intuitive (also called type 1 processes; [3]), and when we ask people to reflect on these processes they will often ‘construct’ plausible answers. Again, they really believe in their answers but nevertheless they are not in line with their actual behaviour. In a recent study, for example, we asked experienced radiologists how they visually studied X-rays in order to reach a diagnosis [4]. Most of them said they used ‘systematic viewing’, ensuring that all parts of the image are carefully inspected in a systematic fashion and thus yielding full coverage of the image. This is also the way they teach visual diagnosis to their students. Interestingly, eye-tracking data showed that this is not what expert radiologists actually do; compared with students, they use more systematic viewing but show *less* coverage because they ignore irrelevant parts of the image. However, this is a highly automated process they are simply not aware of.

My message is rather straightforward. Subjective data are valuable but when researchers are interested in measuring behaviour or cognitive processes it is always best to combine them with more objective data. Too often, what people say is not what people do!
